# Some Considerations to Shew That the Idea of Effecting a Radical Cure of the Bubonocele Ought Not to Be Entirely Abandoned

**Published:** 1803-02-01

**Authors:** 


### *Some Considerations to shew that the Idea of effecting a radical Cure of the Bubonocele ought not to be entirely abandoned.*

THE ingenuity of the most consummate surgeons has for centuries past been exerted in devising some means to accomplish a radical cure of the Bubonocele; with this view castration, the caustic, the punctum aureum, and royal stitch, have been severally practised; but, on account of the incertitude, pernicious and often perilous consequences of the three first of the above operations, they have been most justly exploded from surgery, while the last one has also sunk, perhaps without good reason, into entire disrepute. For, as far as I can judge, i is deserving of much more consideration than mode.n practitioners seem to have bestowed upon it, and is by no means so incapable of being very materially improved but that it may thereby be rendered likely, under certain circumstances, to prove both safe and efficacious.

Ambrose Pare, Wiseman, and many of the ancient authors before them, describe the mode of performing the royal stitch, and, even in their rude way of operating, suc cess was not unfrequently the consequence. Mr. Samuel Sharp, however, appears to be the last modern of any authority, who does not disapprove it, and he was even inclined to think, that it would generally prove successful,

 when when practised with the improvements that he recommended. On the contrary, all the celebrated writers who have flourished since the year 1750, (when his Critical Enquiry was published,) as well in this country as in France and Italy; Pott, Sabbatier, Bertrandi, &c. all avowably proclaim against the propriety of undertaking it.

Although the unskilful manner in which this operation was executed by the ancients, is sufficient to induce us to lay it aside in that form, and the improvements hinted in Mr. Sharp's Critical Enquiry do not, as I conceive, render it so likely to be successful as it might now become in the present refined state of surgical knowledge, yet I cannot agree that Mr. Pott, or any other writer, has adduced objections sufficiently forcible to shew that what is termed the royal stitch ought to be for ever abandoned. Nor does it appear to me, that Mr. Sharp's ideas of the disease and parts affected by it, were erroneous and imperfect, because he was of opinion, that straitening the passage from the abdomen into the scrotum by this operation might prove successful in the radical cure of Bubonocele. It is true, that when Mr. Pott asserts that all its patrons equally betrayed such ignorance, he does not particularly mention Mr. Sharp; nevertheless, since the latter had already several years published his opinion on this subject before the former's Treatise on Ruptures appeared, the severity of the censure must be directed as much against him as any other writer. The author of this little paper does not wish to be esteemed at all critical, nor will he pretend to animadvert on this part of the writings of the illustrious Pott; he only regrets that it does not seem to elucidate the facts upon which some expressions were founded.

Indisputably, it seems to be a very natural idea, that the descent of a hernia must be hindered, by closing the aperture through which the parts protrude; it is on this principle that the operation was done, and that various kinds of trusses have been invented and worn; but, as every body knows, the best made trusses are often inadequate constantly to fulfil the intention, and besides the continual danger of strangulation in which a person lives, whose hernia occasionally comes down, a truss is a very cumbersome thing, and highly inconvenient to such as are young and in the prime of life. Any suggestions therefore which may from time to time be intimated, with a view to procure more certainly a radical cure of the Bubonocele, ought not, most assuredly, to be treated slightingly, and never will be by the unprejudiced. To this part of the profession fession in particular are the following considerations addressed, which are neither inconsistent with the dictates of anatomy, nor the principles of surgery, and as such will at least awaken the minds of some, to scrutinize further the subject.

1st. The hernial sac of a bubonocele, as it is hardly necessary to premise, is formed by an elongated production of the peritoneum, which is pushed down before the protruded viscera either into the groin or scrotum; at its exit through the abdominal ring it is situated over the spermatic cord, upon the anterior part of which and tunica vaginalis it also lies in its course downwards, and is only covered by the common integuments of the groin and scrotum. As it soon becomes adherent to the circumjacent parts after its first protrusion, surgeons are seldom or never able to procure its return.

2dly. Probably it will generally be conceded that a hernial sac is capable of uniting by the adhesive inflammation, just as most other parts of the body do; if any one doubts it, he has only to call to his remembrance cases, where a radical cure has occasionally followed the concretion of the neck of the sac, after inflammation excited by the pressure of the pad of a truss; also others, where its internal surface adheres to the contained viscera.

3dly. The generality of surgeons will, perhaps, allow that it may be united, but not firmly enough to prevent the recurrence of the hernia, and may apprehend that in case it should recur, on account of the altered state of parts, strangulation would take place. To such objections I reply, that reasoning on those radical cures which have occasionally been produced by the pressure of a truss, and commonly have endured through life; and considering the analogy of cases where the sac and contained parts adhere so firmly together, as absolutely to be inseparable without the knife, it seems improbable that the adhesion of the neck of the sac should not be firm enough to prevent a recurrence of the disease; and that the danger apprehended from the altered state of parts, and the chance of another descent, is at least counter-balanced by that peril to which every person, subject to the occasional descent of a hernia, is exposed.

4thly. It is not clear to me, that in performing the operation (which, of course, can never be undertaken but when the parts have been previously returned into the abdomen) that any advantage arose from making the incision the whole length of the hernial sac, as was anciently

 done, done, and which does not appear to have met with the disapprobation of Mr. Sharp; an incision large enough to bring fairly into view the upper part of the sac, close to the abdominal ring, is all that can be rationally useful; for it is a firm union of the neck of the sac alone, that can prevent the hernia from falling down again, and consequently must be the real object to be desired. An incision unnecessarily large, a superfluous number of stitches (as I consider all more than one must be, just as more than one ligature to the mouth of a blood-vessel) can but tend, by their irritation, to augment the inflammation, bring on suppuration, and even gangrene of the parts.

5thly. That the neck of the hernial sac, after it has been fairly exposed by dissection, ought to have one ligature only past through it, immediately before the anterior part of the spermatic cord, and close to the abdominal ring, and that it ought to be tied just as we tie the ligature of an artery. I cannot apprehend that any dangerous constitutional symptoms would be likely to follow such a small wound as would be requisite, or making a ligature on a part of such little importance to the body as a hernial sac; ncr could any but the most unskilful surgeon run any hazard of wounding the spermatic vessels.

6thly. I conceive, that gradually as the success and efficacy of this operation might be evinced, surgeons would be warranted in practising it in almost every case of reducible bubonocele, and even other hernia, for which a truss is at present worn. But, as it would be too bold to advise its performance in such an unbounded degree, we perhaps might feel ourselves authorized at first to make the attempt, after the manner above hinted, only in certain cases, where, as Sharp observes, people are so uneasy as to expose themselves to any measure, in this circumstance, for the hopes of a radical cure. If found once efficacious, it may prove so a hundred times; and, at any rate, I see no reason why, after performing the common operation to relieve a strangulated bubonocele, that opportunity of making a ligature on the neck of the hernial sac should not be taken, to learn whether a radical cure would not oftener follow it than is at present the case.

Lastly, I conclude in the words of Sharp, that it must in its nature be more effectual than the caustic, and less dangerous than the common operation for the Bubonocele, besides it will be much less liable to a relapse, which the usual operation for the Bubonocele is very subject to. I am not unaware of the two unfortunate instances of the operation operation done in France by M. Jean Louis Petit, and *par un de ses Confreres;* nor of the two successful operations of the royal suture done by the above gentleman and a surgeon at Orleans; but as that mode of operating is totally different from the one here suggested, these cases can have but little relation to the question of, whether, when done in the best way, this attempt to relieve from a burdensome disease numbers of our fellow creatures, ought to be for ever abandoned as dangerous and ineffectual?

*January* 8, 1803. S. C.

